# Corrigendum

**DOI:** 10.1002/ehf2.13889

**Published:** 2022-03-10

In the paper by Gustafsson *et al*.,^1^ there is a mistake in the affiliation for Giuseppe Rosano.

Cardiovascular Clinical Academic Group, St George's Hospitals NHS

Trust University of London, London, UK;

RCCS San Raffaele Pisana, Rome, Italy

The correct affiliation is:

Centre for Clinical and Basic Research, Department of Cardiac & Pulmonary Rehabilitation Sciences, IRCCS San Raffaele Pisana, Rome, Italy
